# Counting Spinal Phylogenetic Networks

**DOI:** 10.1007/s11538-026-01624-4

**Published:** 2026-03-18

**Authors:** Andrew Francis, Michael Hendriksen

**Affiliations:** University of New South Wales, Sydney, Australia

**Keywords:** Phylogenetic networks, Enumeration, Expanding covers

## Abstract

Phylogenetic networks are an important way to represent evolutionary histories that involve reticulate processes such as hybridisation or horizontal gene transfer, yet fundamental questions such as how many networks there are that satisfy certain properties are very difficult. A new way to encode a large class of networks, using “expanding covers”, may provide a way to approach such problems. Expanding covers encode a large class of phylogenetic networks, called labellable networks. This class does not include all networks, but does include many familiar classes, including orchard, normal, tree-child and tree-sibling networks. As expanding covers are a combinatorial structure, it is possible that they can be used as a tool for counting such classes for a fixed number of leaves and reticulations, for which, in many cases, a closed formula has not yet been found. More recently, a new class of networks was introduced, called spinal networks, which are analogous to caterpillar trees for phylogenetic trees and can be fully described using covers. In the present article, we describe a method for counting networks that are both spinal and belong to some more familiar class, with the hope that these form a base case from which to attack the more general classes.

## Introduction

Phylogenetic trees and networks are important ways to represent the evolutionary relationships between a set of taxa. While trees describe histories that model evolution together with speciation, networks are able to include other “reticulate” processes such as horizontal gene transfer, hybridisation, or endosymbiosis. The capacity to model these processes comes at the price of complexity, and difficulties in inferring networks from the data, which are typically genetic sequences from extant taxa at the leaves of the network.

In particular, while there are finitely many labelled binary trees on a given number of leaves (exactly $(2n-3)!!$ rooted binary trees on *n* leaves), without further qualification there are infinitely many rooted binary phylogenetic networks. This is one reason why many classes of phylogenetic network have been defined, in attempts to find restrictions that make the problem more mathematically tractable, and hopefully also biologically plausible. A recent summary of many currently described classes can be found in Kong et al. (2022). New classes, however, continue to be defined, and in the present paper we will show that one such new class, the *spinal phylogenetic networks*, can be precisely enumerated.

The class of spinal networks was defined in the context of the study of bijections between phylogenetic networks and covers of a finite set (Francis et al. 2024). They can be defined as those networks for which there is a path from a leaf to the root that includes all non-leaf vertices (a “spine”). In this sense they are direct analogue of *caterpillar trees*, which are defined by this property among phylogenetic trees. Caterpillar trees are an important class of trees for a number of reasons. For instance, they are those trees with a single cherry, and are the least “balanced”, thus providing boundary values for tree balance indices such as the Sackin and Colless indices (Fischer et al. 2023). They also have maximum tree height, and therefore provide worst-case scenarios for algorithms that are dependent on the height of input trees. Consequently, studying combinatorial properties of spinal networks may provide a useful edge-case for the study of phylogenetic networks in more generality.

Enumeration is directly relevant biologically, for many reasons. For example, several biological applications including, for example, Markov Chain Monte Carlo simulations, involve randomly sampling from a given (network) class. In order to properly construct one’s priors, it is necessary to understand the size of the space. Additionally, enumeration is necessary to judge whether certain structures in evolutionary histories may be the result of evolutionary pressures. For instance, in the presence of rapid evolution, such as with viral pathogens, the trees that arise are often close to caterpillar in shape (Fitch et al. 1991; Bush et al. 2000; Bryan et al. 2004). Without knowing the relative size of the class of spinal networks within some more general class, it is difficult to judge whether a similar claim can be made.

We will show in Sect. 4 that the spinal networks can be precisely counted, along with a number of subclasses of spinal networks that have additional properties. Of particular interest are the spinal networks that are also *tree-child*. The tree-child networks (Cardona et al. 2008) (or TCNs) are an important class because they have both properties that make them mathematically workable, as well as properties that may make them possible to reconstruct from data at the leaves.

A considerable amount of recent work has explored the problem of enumeration of tree-child networks. For instance, Fuchs et al. introduced a method based on generating functions to count TCNs with few reticulation vertices (Fuchs et al. 2019), and Cardona and Zhang introduced an algorithm for enumeration of TCNs, allowing TCNs with up to 8 taxa and up to 7 reticulations to be counted (Cardona and Zhang 2020). A range of approaches have also been taken to enumerate classes of phylogenetic networks asymptotically, including tree-child networks (McDiarmid et al. 2015; Fuchs et al. 2019, 2021). Despite this, no closed formula is known for counting TCNs on *n* taxa with *k* reticulations in general.

It is to be hoped that counting these classes of spinal networks will provide a valuable base from which to approach harder open problems, such as counting tree-child networks more generally. For instance, one conjecture is that the number $|TC_{n,k}|$ of binary tree-child networks with *k* reticulations and *n* leaves is given by the recursion (Pons and Batle 2021, Eq. 13):

1 $$\begin{aligned} (n - k)|TC_{n,k}|=(n + 1 - k)(n - k) |TC_{n,k-1}| + n (2n + k - 3) |TC_{n-1,k}|. \end{aligned}$$

This conjecture is due to a hypothesised mapping from tree-child networks to a certain class of words. The spinal networks that are tree-child may be able to play a role as a base case from which to identify a recursion for the number of tree-child networks. Note: while this paper has been under review, a proof of the above conjecture using a different approach has been announced (Lin et al. 2026).

This paper will count spinal networks as well as some subclasses of them, using the bijection between labellable phylogenetic networks and expanding covers of a finite set (Francis and Steel 2023). These subclasses of spinal networks include tree-child networks, binary networks, stack-free networks and a novel class termed fully tree-sibling networks.

## Preliminaries

### Networks and Covers

This article defines phylogenetic networks as follows.

#### Definition 2.1

A *(rooted) phylogenetic network* on leaf-set *X*, with $|X|=n$, is a directed acyclic graph whose vertex set satisfies the following:

- There is one vertex of in-degree 0, called the *root*;
- There are *n* vertices of in-degree 1 and out-degree 0, called *leaves* and labelled by elements of *X*; and
- All other vertices, the *internal* vertices, can have any in-degree or out-degree $\ge 1$.

Note that this definition is more broad than many definitions of phylogenetic networks, in that it permits so-called *degenerate* vertices, which have either in-degree and out-degree 1, or both in-degree and out-degree strictly greater than 1. Networks containing a degenerate vertex are also called *degenerate*, and a network containing no degenerate vertex is *non-degenerate*. However, with the exception of Sect. 4.6.1, we do not permit parallel arcs, that is, multiple arcs between the same pair of vertices. A network is considered *binary* if all internal vertices have total degree exactly 3, and the root has out-degree 2. Note that, by definition, a binary network is non-degenerate. The recursive formula conjectured to count tree-child networks (Eq. (1)) is for binary networks.

A vertex that has in-degree less than 2 is called a *tree vertex* (including the root and the leaves), while vertices of in-degree greater than or equal to 2 are called *reticulation vertices*. If there is an arc (*u*, *v*), then *u* is termed a parent of *v*, and *v* is termed a child of *u*. A pair of vertices *v* and *w* are termed *siblings* if both *v* and *w* share a parent *u*. A *path* in a phylogenetic network is a sequence of distinct vertices $v_1,\dots ,v_t$ such that for each $1\le i<t$, $(v_i,v_{i+1})$ is an arc in *N*; in this case we say this is a path *from* $v_1$ *to* $v_t$. For a phylogenetic network *N*, define the *height* *h*(*N*) of *N* to be the length of the longest path in *N*.

In this paper we will be making use of the bijection between the class of *labellable* networks, and “expanding covers” of finite sets (Francis and Steel 2023). Recall the following definitions.

#### Definition 2.2

Denote by [*m*] the set $\{1,\dots ,m\}$. A *cover* of [*m*] is a collection of subsets $\mathcal {C}=\{C_1,\dots ,C_{|\mathcal {C}|}\}$ (with $|\mathcal {C}|$ denoting the number of subsets in $\mathcal {C}$) such that the union

$$\begin{aligned} \bigcup _{i=1,\dots ,|\mathcal {C}|} C_i = [m]. \end{aligned}$$

If some element $c \in [m]$ is contained in *k* sets in $\mathcal {C}$ (that is, $c \in C_{i_1},\dots ,C_{i_k}$) for some $\{i_1,\dots ,i_k\} \subseteq [|\mathcal {C}|]$) we say that *c* *appears k times* in $\mathcal {C}$. If *c* appears *k* times in $\mathcal {C}$ for some $k>0$, we may say that *c* *appears* in $\mathcal {C}$ with no quantifier.

The labellable networks are a large class that contains all the classes discussed below, and many commonly studied classes can themselves be described in terms of a bijection with expanding covers that satisfy certain additional conditions (Francis et al. 2024).

Given a phylogenetic network whose leaves are labelled by an ordered set $[n]:=\{1,\dots ,n\}$, there is an algorithm, which we term the *labelling algorithm*, for labelling the remaining non-root vertices, that proceeds by identifying the unlabelled vertex with the least set of children, according to the lexicographic order on sets:

$$\begin{aligned} A \prec B \text { if } A \subseteq B \text { or } \min (A \backslash (A \cap B)) < \min (B \backslash (A \cap B)). \end{aligned}$$

The labelling algorithm generalises a similar one for phylogenetic trees (Erdős and Székely 1989).

Explicitly, the algorithm takes as input a rooted phylogenetic network *N* on *n* leaves that are labelled by the set $\{1,\dots , n\}$, and proceeds as follows (Francis and Steel 2023, Algorithm 1):

1. Initialise by setting $i=0$ and *I* to be the set of unlabelled internal vertices of *N*.
2. While *I* is non-empty, do the following:
  - Set *A* to be those vertices in *I* whose children are all labelled.
  - Choose a $v\in A$ such that the set of labels of its children is minimal under the lexicographic order.
  - Assign *v* the label $n+i+1$, and remove *v* from *I*.
  - Increment *i* by 1.
3. Output the network *N* with all non-root vertices labelled by integers $\{1,\dots ,n+i+1\}$.

A phylogenetic network is said to be *labellable* if the labelling algorithm above is deterministic, that is, if at each choice of which vertex *v* to label next (step 2(b)), there is a *unique* unlabelled vertex with the least set of labels of children. Labellable networks can be characterised in terms of certain forbidden substructures (Francis and Steel 2023, Thm 3.3), but for our purposes the important characterisation is that they are in bijection with the set of *expanding covers*:

#### Definition 2.3

*( * Francis and Steel (2023)) A cover $\mathcal {C}$ of [*m*] is *expanding* if, for $n = m - |\mathcal {C}| + 1$, it satisfies:

1. No element of [*n*] appears more than once in $\mathcal {C}$, and
2. For $i = 1, \dots , |\mathcal {C}|$, the cover contains at least *i* subsets of $[n + i - 1]$.

For example, let $\mathcal {C}=\{\{2,3\},\{1,5\},\{5\},\{4,7\},\{5,6\},\{5,7,9\},\{8,10\} \}$. This is a cover of [10], so $m=10$ and $|\mathcal {C}|=7$. Hence $n=10-7+1=4$, and one can observe that no element of [4] is repeated, and for each of $i = 1,\dots ,7$, there are at least *i* subsets of $[i+3]$. Hence $\mathcal {C}$ is an expanding cover. In fact, we have the following theorem.

#### Theorem 2.4

( Francis and Steel (2023), Theorem 4.4) The set of labellable phylogenetic networks on *n* leaves with *m* vertices is in bijection with the set of expanding covers on [*m*] with $m-n+1$ subsets in the cover.

**Fig. 1:**
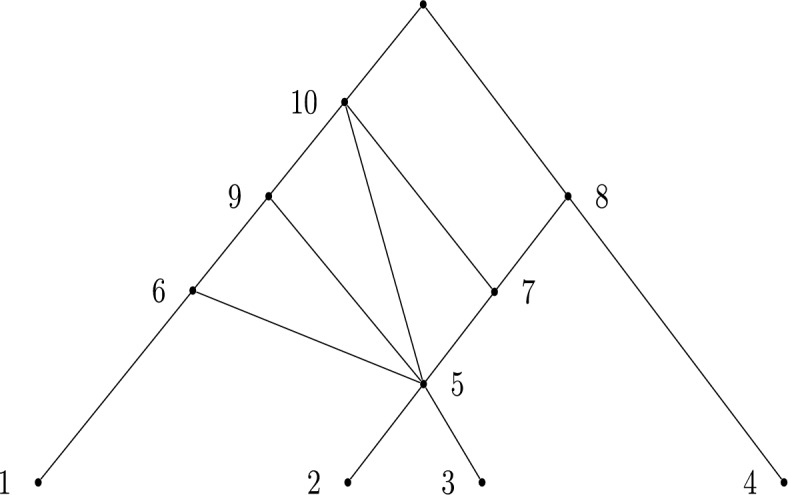
An example of a labellable network. This network corresponds to the expanding cover $\{\{2,3\},\{1,5\},$ $\{5\},\{4,7\},\{5,6\},\{5,7,9\},\{8,10\}\}$, written in labelling order. This is a cover of [*m*] for $m=10$, and since $|\mathcal {C}|=7$, we see $n=m-|\mathcal {C}|+1=10-7+1=4$, corresponding to the four leaves

In particular, let *N* be a labellable network mapped to some expanding cover $\mathcal {C}$ under this bijection. This leads to the natural intepretation of the value *n* as being the number of leaves in *N*, the value *m* as the number of non-root vertices and the value $|\mathcal {C}|$ as the number of internal vertices (including the root). We provide an example of a labellable network with associated expanding cover $\{\{2,3\},\{1,5\},$ $\{5\},\{4,7\},\{5,6\},\{5,7,9\},\{8,10\} \}$ in Figure 1.

The labelling algorithm on vertices in a labellable network defines a bijection between the non-leaf vertices in the network, and the sets in the expanding cover for the network. This bijection maps each such non-leaf vertex *v* to the set of labels of its children. This bijection means that the sets in the expanding cover inherit an ordering from the ordering on the labels of the non-leaf vertices (where the root vertex is treated as maximal). We call this ordering on the sets in the expanding cover, the *labelling order* for the cover.

The expanding cover associated with the network in Figure 1, and given above, is written in labelling order. Because $n=4$ here, the set $\{2,3\}$ corresponds to the label 5 (the children of the vertex labelled 5 have labels 2 and 3), the set $\{1,5\}$ corresponds to the label 6, and so on. The labelling order accentuates the connection to expanding covers, as one can see the first *i* sets are all contained in $[n+i-1]$ ($[i+3]$ in this case). In the reverse direction, satisfying the expanding property ensures that a network can be built deterministically from a cover (see Francis and Steel (2023) for details).

We can now move to the main topic of this paper, which is the class of *spinal networks*.

### Spinal Networks

We define a *spine* in a network *N* to be a sequence of vertices $(\ell =v_0, v_1,\dots ,v_t=\rho )$, where $\ell$ is a leaf, $\rho$ the root, $\{v_1,\dots ,v_t\}$ contains all non-leaf vertices, and for each $0\le i<t$ there is an arc $(v_{i+1},v_i)$. Informally, a spine is a (reversed) path from a leaf to the root that traverses all internal vertices.

**Fig. 2:**
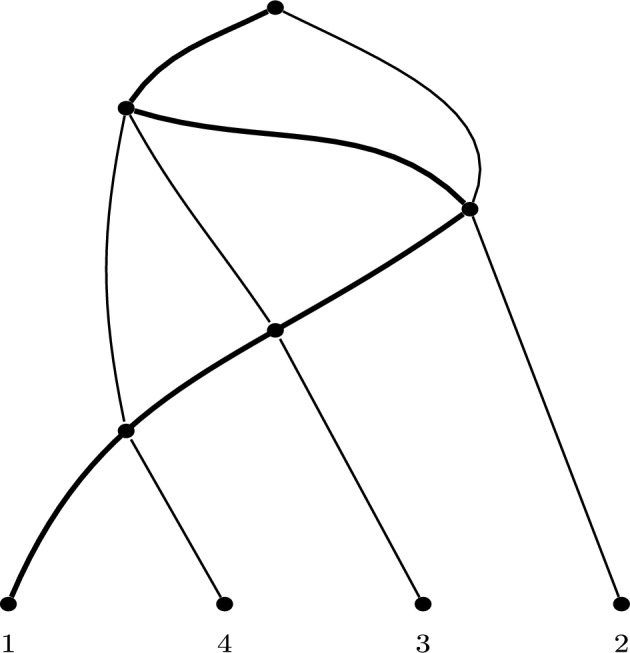
Example of a spinal network, with spine shown in bold passing through all vertices from the root to a leaf

We call a network *spinal* if it has a spine. Note that if a network has a spine, then the spine is unique up to choice of leaf (see Lemma 3.1). An example of a spine in a network is given in Figure 2.

Spinal networks may be characterised by their covers, as follows.

#### Theorem 2.5

(Francis et al. (2024), Theorem 8.1) A network is spinal if and only if its cover $\mathcal {C}$ has exactly *i* subsets of $[n + i - 1]$, for each $i = 1, \dots , |\mathcal {C}|$.

Compare this characterisation to Definition 2.3 — specifically that the words “at least” are replaced by “exactly”. The following Corollary has been noted in the proof of (Francis et al. 2024, Theorem 8.1).

#### Corollary 2.6

If $\mathcal {C}=\{C_1,\dots ,C_{|\mathcal {C}|}\}$ is the expanding cover for a spinal network, then for each $i=2,\dots ,|\mathcal {C}|$, the set $C_i$ must contain $n+i-1$.

This means that the cover for any spinal network has the structure:

2 $$\begin{aligned} \big \{\{\text {some leaves}\},\{n+1,\dots \},\{n+2,\dots \},\dots ,\{n+|\mathcal {C}|-1,\dots \}\big \}, \end{aligned}$$

in which the elements in each set of the form $\{n+i,\dots \}$ are at most $n+i$.

We will also consider several other classes of phylogenetic networks, including one novel one. We present the existing classes first.

#### Definition 2.7

*( * Cardona et al. (2008)) *Tree-child* networks are phylogenetic networks for which every non-leaf vertex has at least one child that is a tree vertex.

#### Definition 2.8

A network is *stack-free* if every child of a reticulation vertex is a tree vertex.

We also define a new class of phylogenetic network.

#### Definition 2.9

A network *N* is *fully tree-sibling* if and only if every sibling of a reticulation vertex is a tree vertex.

**Fig. 3:**
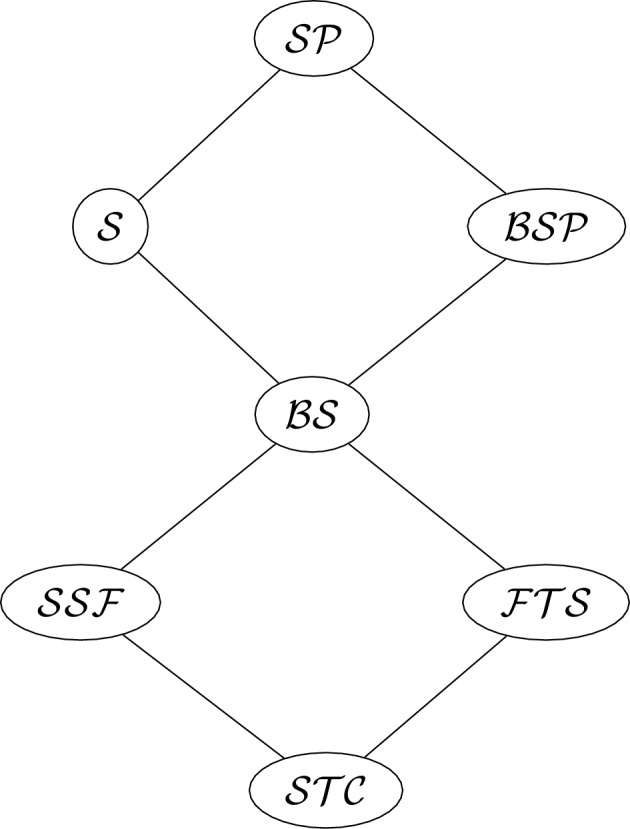
The Hasse diagram for types of spinal networks considered in this paper under the inclusion order. Note that, in this case, the classes with two parents are exactly the intersection of those parents

Note that a network in each of these three classes of networks may be degenerate or non-binary, but we will only consider binary (and hence non-degenerate) networks in these classes in the present article. Further note that fully tree-sibling networks are a subclass of the well-known tree-sibling networks, as tree-sibling networks only require each reticulation vertex to have *at least one* sibling that is a tree vertex, rather than *every* sibling. These three classes will be further described in the relevant sections. In Table 1 we present notation for each class of spinal networks described in this article, for use in the Hasse diagram describing class inclusion, Figure 3. When this notation is used later, it will usually have some subscripts to restrict, for example, the number of leaves and number of reticulations. We also note that in this article we do not count the class of all spinal networks with parallel arcs permitted (with notation $\mathcal{S}\mathcal{P}$ in the table and diagram) as without further restriction this class is infinite, even when limited to a certain number of leaves and reticulations. The table uses brackets to indicate the letters used in the notation, for ease of reference.

**Table 1:** Notation for the classes described in this article, together with a reference to the theorem in the this article that enumerates this class. Enumeration of $\mathcal{S}\mathcal{P}$ is not covered in this article as the class is infinite even when limited to a certain number of leaves and reticulations

**Notation**	**Class description**	Theorem
$\mathcal{S}\mathcal{P}$	(S)pinal networks, (P)arallel arcs permitted	-
$\mathcal {BSP}$	(B)inary (S)pinal networks, (P)arallel arcs permitted	4.26
$\mathcal {S}$	(S)pinal networks, no parallel arcs	4.2
$\mathcal{B}\mathcal{S}$	(B)inary (S)pinal networks, no parallel arcs	4.27
$\mathcal {SSF}$	Binary (S)pinal (S)tack-(F)ree networks, no parallel arcs	4.18
$\mathcal {FTS}$	Binary spinal (F)ully (T)ree-(S)ibling networks, no parallel arcs	4.24
$\mathcal {STC}$	Binary (S)pinal (T)ree-(C)hild networks, no parallel arcs	4.11

We now turn to some elementary properties of spinal networks.

## Properties of Spinal Networks

### Lemma 3.1

Let *N* be a spinal network. Then the spine is unique up to the choice of the initial leaf.

### Proof

Any spine must contain only a single leaf, $v_0$, as a leaf does not have any children. Hence all other vertices in the sequence for the spine must be internal, except for $v_t$, the root. Suppose we have two distinct spines, $S_1 = (v_0,\dots ,v_t)$ and $S_2=(w_0,\dots ,w_t)$ that first differ at the *i*-th entry for $0<j<t$, i.e. $v_i \ne w_i$. As both spines must contain all internal vertices, $v_i$ must then appear subsequently to $w_i$ in $S_2$, say at $w_j$. Similarly, $w_i$ must appear subsequently to $v_i$ in $S_1$, say at $v_k$. It follows that $S=(w_i,\dots ,w_j=v_i,\dots ,v_k=w_i)$ is a sequence of vertices such that each entry except the initial $w_i$ has an arc from the next vertex to itself. Equivalently, the reverse of the sequence *S* (i.e. $(w_i=v_k,\dots ,v_i=w_j,\dots ,w_i)$) is a sequence of directed arcs that starts and finishes at the same vertex $w_i$. This contradicts the fact that *N* is a directed acyclic graph, and so the spine must be unique up to choice of the first leaf. $\square$

Spinal networks can be seen as a generalisation of caterpillar trees. For instance, the only spinal networks that are also phylogenetic trees are precisely the set of caterpillar trees. Additionally, binary caterpillar trees have maximal height among binary phylogenetic trees, and we have the following similar properties for spinal networks: that among all phylogenetic networks with a fixed number of internal vertices and in particular, among all binary phylogenetic networks with *n* leaves and *k* reticulations, spinal networks are precisely those that have maximal height.

### Lemma 3.2

Among all phylogenetic networks with a fixed number of internal vertices, spinal networks are precisely those that have the maximal height. In particular, among all binary phylogenetic networks with *n* leaves and *k* reticulations, spinal networks are precisely those that have maximal height.

### Proof

If a network has *m* internal vertices, then the longest path possible in the network has length $m+1$, and goes from the root to a leaf. This defines a spine (reverse), and so any network with maximal height must be spinal. In contrast, a network whose longest path is less than $m+1$ does not have a path that includes all internal vertices, and so is not spinal.

The second claim follows immediately, by observing that every binary phylogenetic network with *n* leaves and *k* reticulations has the same number of internal vertices, namely $n+2k-2$. $\square$

## Counting Classes of Spinal Networks

We will now proceed to leverage the expanding cover interpretation of labellable phylogenetic networks, together with the naturally restrictive characterisation of spinal networks, to enumerate various classes of spinal networks. With the exception of the class of all spinal networks, which we will deal with first, these enumerations will follow a reasonably similar structure — first characterising the vertices in a spine of a spinal network of some particular class, then creating a surjection based on this characterisation into a nice set-theoretic class, and then modifying this result to fully count the relevant subclass of spinal networks. We will give a more complete overview of this structure in Sect. 4.2.

In Sect. 4.1, phylogenetic networks are permitted to be degenerate and non-binary, but in subsequent sections every network will be binary, non-degenerate, and spinal. We begin by noting some helpful lemmas.

For some class of networks *R*, define the related class of *network shapes in* *R*, denoted *S*(*R*), to be the set of equivalence classes of *R* under the equivalence relation $\sim _S$, where $N_1 \sim _S N_2$ are equivalent if there exists a permutation on the labels of the leaves of $N_1$ for which we obtain a network isomorphic to $N_2$.

We will also need the notion of *cherry reduction* for a binary spinal network shape *N*. If a vertex has only one parent, denote the parent of a vertex *x* by *p*(*x*). A pair of leaves (*x*, *y*), who by definition only have one parent each, is a *cherry* in a network if *x* and *y* have the same parent $p(x)=p(y)$. If *N* is not the unique tree on two leaves, reducing a cherry (*x*, *y*) in a network is the action of deleting the two vertices *x* and *p*(*x*) and the three arcs (*p*(*x*), *x*), (*p*(*p*(*x*)), *p*(*x*)) and (*p*(*x*), *y*) and adding a single arc (*p*(*p*(*x*)), *y*). Otherwise, if *N* is the tree on two leaves, simply replace *N* by the tree on the single leaf *y*. Note that *p*(*p*(*x*)) is well-defined in the former case, as *N* is a binary network, so as *p*(*x*) has two child vertices it can only have one parent vertex.

We also define a *re-cherrying* operation on a binary spinal network shape, in which one of the (up to) two leaf arcs furthest from the root is subdivided, and a new leaf is attached to the resulting degree-2 vertex. This is a special case of an operation called “cherry expansion” that has been defined (in different ways) in several places (Katharina et al. 2013; Landry et al. 2022). Note that in the context of binary spinal network shapes, the only possible cherry that can appear would be on the children of the internal vertex furthest from the root, so cherry reduction and re-cherrying are inverse operations for binary spinal network shapes (note that these are not inverse operations for more general phylogenetic network shapes).

### Lemma 4.1

Let *R* be a class of binary spinal networks that is closed under permutations of leaf labels, cherry reductions and re-cherrying. If $R_{n,k}$ denotes the subclass of *R* on *n* leaves with *k* reticulations, then

$$\begin{aligned} |R_{n,k}|=n!|S(R_{n,k})|-\frac{n!}{2}|S(R_{n-1,k})|. \end{aligned}$$

### Proof

Note that for any fixed spinal network *N*, there exists a unique path from a leaf to the root up to choice of the initial leaf if the first internal vertex of the path has two leaf children. Therefore, for each leaf $\ell$ that is a child of the *i*-th internal vertex, any permutation on the labels of the leaves that does not have $\ell$ as a fixed point will result in a different network that has the same network shape. Denote the subclass of $S(R_{n,k})$ with *i* children of the first vertex by $L_i$, noting that $|S(R_{n,k})|=|L_1|+|L_2|$. It follows that if *N* has only one child of the first internal vertex (that is, *N* has no cherries), the equivalence class of network shapes in $L_1$ containing *N* will contain *n*! networks (because each permutation of the *n* leaves will give a distinct network). However, if *N* has two children of the first internal vertex, the equivalence class of network shapes in $L_2$ will contain *n*!/2 networks (as a consequence of the fact that the permutation swapping these labels of these two children does not result in a new network). It follows that

$$\begin{aligned} |R_{n,k}|=n!|L_1|+\frac{n!}{2}|L_2|=n!(|S(R_{n,k})|-|L_2|)+\frac{n!}{2}|L_2|=n!|S(R_{n,k})|-\frac{n!}{2}|L_2|. \end{aligned}$$

We finally note that there exists a straightforward bijection between $L_2$ and network shapes in $S(R_{n-1,k})$. This is obtained by cherry reduction of the unique cherry of networks in $L_2$ (which must exist, as there are two leaf children of the first internal vertex) in the forward direction to obtain a network shape in $S(R_{n-1,k})$, and the unique possible re-cherrying in the inverse direction. The result follows. $\square$

### The Class of all Spinal Networks

Denote the cardinality of the class of spinal networks on *n* leaves with covers of size $|\mathcal {C}|$ by $\mathcal {S}_{n,|\mathcal {C}|}$. Note that, in contrast to all other results in this manuscript, in this section we are not restricting these networks to be binary.

The restrictions on the covers for a spinal network make it possible to enumerate the whole class quite directly, as in the following theorem. The proof proceeds by assigning each leaf to a set, and then counts the ways multiplicities of other vertex labels may be distributed among the sets.

#### Theorem 4.2

Let $n, |\mathcal {C}|\ge 1.$ Then, the cardinality of the class of spinal networks on *n* leaves with covers of size $|\mathcal {C}|$ is

$$\begin{aligned} \mathcal {S}_{n,|\mathcal {C}|} = 2^{\left( {\begin{array}{c}|\mathcal {C}|-1\\ 2\end{array}}\right) }(|\mathcal {C}|^n - (|\mathcal {C}|-1)^n), \end{aligned}$$

where for $|\mathcal {C}|=1,2$ we set $$\left( {\begin{array}{c}|\mathcal {C}|-1\\ 2\end{array}}\right) = 0$$.

#### Proof

Note first that by virtue of being a spinal network, the structure of the expanding cover under the labelling order is very restricted. In particular, by Corollary 2.6 (see also Eq. (2)), it is not necessary to consider the labelling order explicitly. This is due to the fact that the position of a set in the order is made clear by considering whether the largest element in the set is

- a leaf, in which case the set is $C_1$, or
- the element $n+i-1$ for $i>1$, in which case the set is $C_i$.

We now assign the leaves to the sets in the cover. The leaves may be assigned to any set in any order, but each must be assigned to a single set, and with the caveat that $C_1$ must be non-empty. There are therefore

3 $$\begin{aligned} |\mathcal {C}|^n - (|\mathcal {C}|-1)^n \end{aligned}$$

options for doing this (all options, minus those for which $C_1$ is empty).

Then, for each $i \in 2,\dots ,|\mathcal {C}|$, each element $n+i-1$ must be contained in the set $C_i$ and no other $C_s$ for $s<i$. For $j=i+1,\dots ,|\mathcal {C}|$, the element $n+i-1$ either is or is not contained in set $C_j$, for a total of $2^{|\mathcal {C}|-i}$ possibilities for each *i*. Multiplying over all possible *i*, we obtain

4 $$\begin{aligned} \prod _{i=2}^{|\mathcal {C}|} 2^{|\mathcal {C}|-i} = 2^{\sum _{j=0}^{|\mathcal {C}|-2} j} = 2^{\left( {\begin{array}{c}|\mathcal {C}|-1\\ 2\end{array}}\right) }. \end{aligned}$$

Multiplying expressions (3) and (4) together gives the result in the proposition. $\square$

An example of the process described in the above proof is given in Example 4.3.

#### Example 4.3

Suppose *N* is a spinal network with $n=4$ and $|\mathcal {C}|=5$. Then by Corollary 2.6 the cover for *N* is of form

$$ \big \{\{\text {some leaves}\},\{\textbf{5},\dots \},\{\textbf{6},\dots \},\{\textbf{7},\dots \},\{\textbf{8},\dots \}\big \}. $$

Choosing leaves $\{1,4\}$ for the first set, and labels to add to other sets less than the bold element, an example could be:

$$ \big \{\{1,4\},\{\textbf{5},3\},\{\textbf{6},2\},\{\textbf{7},6,5\},\{\textbf{8},7\}\big \}. $$

**Fig. 4:**
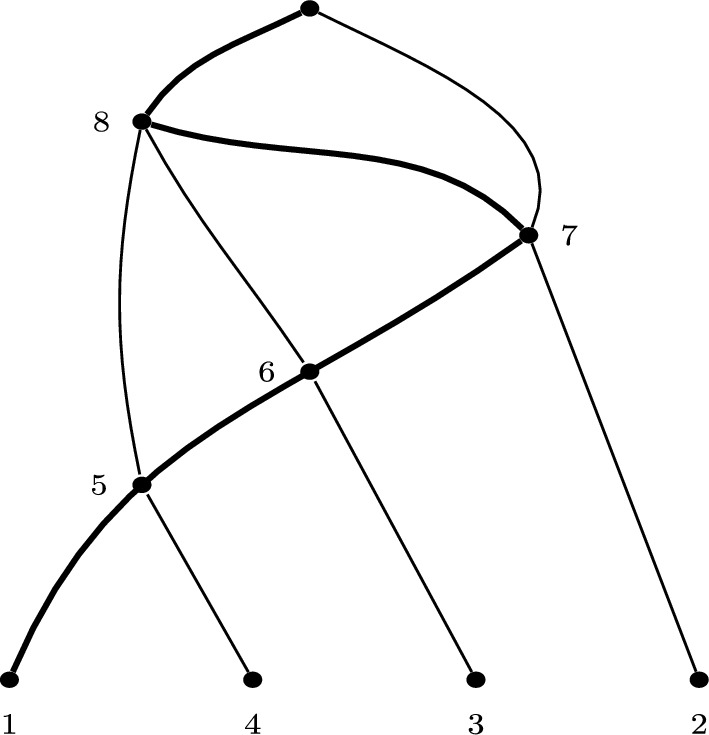
The labellable network with $n=4$ and $|\mathcal {C}|=5$, corresponding to Example 4.3, also shown in Figure 2 without internal labels. A spine is shown in bold (unique up to choice of leaf 1 or 4, as per Lemma 3.1)

This has corresponding spinal network as shown in Figure 4. A spine for the network is given by a choice of leaf from the first set followed by the vertices corresponding to the increasing bold vertex labels, and is shown in bold in the Figure.

**Table 2:** Values for $\mathcal {S}_{n,|\mathcal {C}|}$ where $1 \le n,|\mathcal {C}| \le 8$, calculated using Proposition 4.2

$|\mathcal {C}| \backslash n$	**1**	**2**	**3**	**4**	**5**	**6**	**7**	**8**
**1**	1	1	1	1	1	1	1	1
**2**	1	3	7	15	31	63	127	255
**3**	2	10	38	130	422	1330	4118	12610
**4**	8	56	296	1400	6248	26936	113576	471800
**5**	64	576	3904	23616	134464	737856	3951424	20805696
**6**	1024	11264	93184	687104	4762624	31775744	206654464	1319926784
**7**	32768	425984	4161536	36208640	295927808	2326298624	17812914176	133863342080
**8**	2097152	31457280	354418688	3554672640	33472643072	303027978240	2670951661568	23094708142080

We provide some small values for $\mathcal {S}_{n,|\mathcal {C}|}$ in Table 2. Note that these should not be directly compared to subsequent tables in the present article, as the inputs for this formula are the number of leaves and the size of the cover, whereas all subsequent tables take their inputs to be the number of leaves and *the number of reticulations*. Further, note that the first row of the table below represents covers with a single set, and therefore correspond to the star tree (of which there is only one for each *n*).

### A Brief Overview of Some Subsequent Proofs

Results in each of the following 3 sections (Sections 4.3, 4.4 and 4.5) follow a similar method of proof, which we will describe informally for ease of following the proofs.

In each case, we will specifically count the binary spinal network shapes with no parallel arcs that belong to some given class (tree-child, stack-free or fully tree-sibling) and have *n* leaves and *k* reticulations. Fix some given class of the three options (tree-child, stack-free or fully tree-sibling) and denote the subset of this class with *n* leaves and *k* reticulations by $\mathcal{T}\mathcal{S}(n,k)$. We will refer to the path along the spine, including arcs, as the *spinal path*, which will start at a leaf and end at the root. We note here that due to the lack of parallel arcs this is uniquely defined up to the choice (between up to 2 options) of the initial leaf, per Lemma 3.1. Then, each internal vertex *v* along the spinal path may be labelled according to which of the three mutually exclusive properties it has:

1. *v* has a (L)eaf child that is not on the spine (*L*),
2. *v* is the *i*-th (R)eticulation vertex counting along the spine from the leaf ($R_i$), or
3. *v* is the (P)arent of the *i*-th reticulation vertex, via an arc not in the spinal path ($P_i$).

We can therefore encode each network shape in $\mathcal{T}\mathcal{S}(n,k)$ as a finite sequence of the letters $\{L,R_1,\dots ,R_k,P_1,\dots ,P_k\}$ containing $n-1$ *L*’s and one copy of each of $R_1,\dots ,R_k$ and of each of $P_1,\dots ,P_k$, with certain restrictions on the order in the sequence. We will call this sequence an *LRP-sequence*. In particular, the LRP-sequence for any spinal network must obey the following three rules:

(R1): If $j>i$, then $R_j$ must appear subsequently to $R_i$;
(R2): $P_i$ must appear subsequently to $R_i$; and
(R3): $P_i$ must not immediately follow $R_i$.

**Fig. 5:**
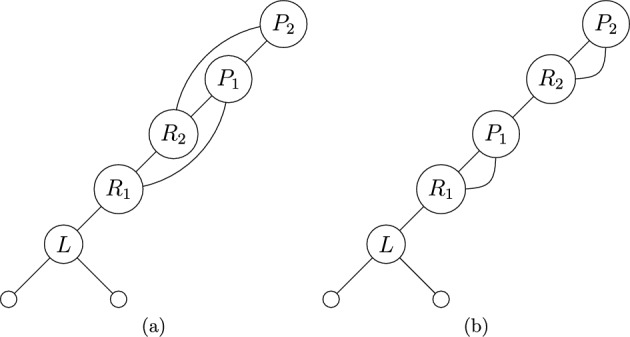
(a) An example of a binary spinal network that has an associated sequence, $(L,R_1,R_2,P_1,P_2)$ that adheres to (R1-3). (b) An example of a binary spinal network that has an associated sequence $(L,R_1,P_1,R_2,P_2)$ that fails (R3), as $P_1$ immediately follows $R_1$, and $P_2$ immediately follows $R_2$. Note that this causes two pairs of parallel arcs

We note that R3 follows from the requirement that our networks have no parallel arcs, as a subsequence of the form $R_iP_i$ would indicate a reticulation vertex that is connected to its parent vertex both along the spinal path and through an arc that is not in the spinal path - that is, a pair of parallel arcs. We will later briefly consider networks with parallel arcs in Sect. 4.6.1, but will not follow the present proof structure. An example of a binary spinal network with an LRP-sequence that follows (R1-R3), together with an example that fails (R3), are presented in Figure 5. The rules (R1-2) follow immediately from the definitions of *R*- and *P*-vertices.

The specific network class will then impose additional restrictions. For instance, in a stack-free network a reticulation vertex must not be a child of another reticulation vertex, which in this encoding would require that there is no subsequence of the form $R_iR_{i+1}$. We note that while we consider 3 possible additional rules in the present article, it may be interesting to consider other additional rules or variations on those we present, and consider what class of binary spinal networks these may correspond to. This may be helpful in future exploration of binary spinal networks.

**Fig. 6:**
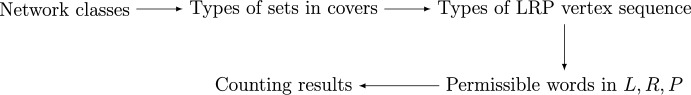
Workflow for counting arguments involving the LRP classification of vertices in various classes of spinal network shapes, and subsequently all networks in the class

The next step involves mapping valid LRP-sequences to some other known set for which the enumeration is already known. Finally, we then apply Lemma 4.1 to count the number of networks, rather than just network shapes. This workflow is summarized in Figure 6.

### The Class of Binary Spinal Tree-Child Networks

In this section, all networks are assumed to be non-degenerate. That is, every non-root internal vertex has out-degree 1 or in-degree 1, but not both. Recall the definition for tree-child networks (TCNs), which is given in Definition 2.7.

#### Theorem 4.4

(Francis et al. (2024), Theorem 4.1) Tree-child networks are in bijection with expanding covers for which each set contains an integer that appears exactly once in the cover.

Let *N* be a binary spinal TCN with associated expanding cover $\mathcal {C}$. As mentioned in the proof of Theorem 4.2, since *N* is spinal, there exists a simple way to determine the position of a set in the labelling order by considering whether the largest element in the set is a leaf (in which case the set is $C_1$) or the element $n+i-1$ for $i>1$, in which case it is $C_i$. The covers of non-degenerate networks can be characterised in the following way:

#### Theorem 4.5

( Francis and Steel (2023), Theorem 5.1) Let *N* be a labellable phylogenetic network with cover $\mathcal {C}$ in labelling order $C_1,\dots ,C_{|\mathcal {C}|}$. Then *N* is non-degenerate if and only if, for each $i \ge 1$:

- If $|C_i| > 1$, then $(n + i )$ appears at most once in $\mathcal {C}$; and
- If $|C_i| = 1$, then $(n + i )$ appears more than once in $\mathcal {C}$.

This can become a characterisation of binary networks with some small modifications which we present here, using a restriction on subset sizes and tighter restrictions on the number of appearances of $(n+i)$ in $\mathcal {C}$.

#### Theorem 4.6

Let *N* be a labellable phylogenetic network with cover $\mathcal {C}$ in labelling order $C_1,\dots ,C_{|\mathcal {C}|}$. Then *N* is binary if and only if all sets in $\mathcal {C}$ have size 1 or 2, and, for each $i \ge 1$:

- If $|C_i | = 2$, then $(n + i )$ appears once in $\mathcal {C}$; and
- If $|C_i| = 1$, then $(n + i )$ appears twice in $\mathcal {C}$.

Together with the condition that a network is spinal, this tightly limits our options for constructing an expanding cover for a binary spinal TCN. For instance, if $|C_i | = 2$, then $(n + i )$ appears once in $\mathcal {C}$, and by the spinal condition we know that it specifically must be contained in $C_{i+1}$. In fact, we can characterise the sets appearing in an expanding cover for a binary spinal TCN even further, as follows (this will be re-cast in Theorem 4.9 into the framework described in Sect. 4.2, to help characterising possibilities).

#### Theorem 4.7

Let *N* be a binary spinal TCN with expanding cover $\mathcal {C}$. Then $C_1$ consists of either one or two leaves, and for $i>1$ each set $C_i\in \mathcal {C}$ falls into one of the following three mutually exclusive categories:

1. It is part of a consecutive pair of sets $C_i$, $C_{i+1}$ such that $C_{i}=\{n+i-1\}$ and $C_{i+1}$ contains $n+i$ and a leaf; or
2. $C_i$ contains $n+i-1$ and a leaf, and is not the second set in a Category 1 pair (i.e $C_{i-1} \ne \{n+i-2\}$); or
3. $C_i$ uniquely contains $n+i-1$ and some element $n+j$ for $0<j<i-1$ so that $C_j$ is a singleton set (and so is the first element of a pair in Category 1).

Indeed, for each pair of sets in Category 1, there is a corresponding set in category 3.

#### Proof

Clearly $|C_1|=1$ or 2 since *N* is binary, and the elements of $C_1$ must be leaves, because $\mathcal {C}$ is an expanding cover. Henceforth we will assume that $i>1$. We will first show that if $|C_i|=1$, then $C_{i+1}$ has precisely the properties required to be in Category 1. Subsequently, we will show that if $|C_i|=2$ and is not part of a consecutive pair in Category 1, then $C_i$ must be in Category 2 or 3.

**Category 1:**

We now show that for $i>1$, if $|C_i|=1$ (so that $C_i=\{n+i-1\}$), then $C_{i+1}$ contains $n+i$ and a leaf.

Since $|C_i| = 1$, then $n + i$ appears twice in $\mathcal {C}$, because *N* is binary, by Theorem 4.6. As *N* is spinal, $C_{i+1}$ must be one of the two sets that contain $n+i$. However, as *N* is a TCN, by Theorem 4.4, $C_{i+1}$ must contain an integer that appears only once in the cover, so $C_{i+1}$ cannot be a singleton. But the other element of $C_{i+1}$ cannot be $n+j-1$ for any $1<j<i+1$ (i.e. none of $n+1,\dots ,n+i-1$), as by the spinal property these are certainly already contained in $C_j$. It follows that the other element of $C_{i+1}$ must be a leaf.

**Category 2:**

We now suppose that $i>1$ and $|C_i|=2$. As *N* is spinal, $C_i$ contains $n+i-1$. If the other element of $C_i$ is a leaf, then $C_i$ is in Category 2.

**Category 3:**

It therefore suffices to show if $C_i$ is *not* in Category 2, then it must be in Category 3. That is, we need to show that the other element of $C_i$ — which must be $n+j$ for $0<j<i-1$ as it is not a leaf — has the property that the corresponding $C_j$ is a singleton (and so the first element of a pair in Category 1).

As *N* is a spinal network, the other element $n+j$ of $C_i$ must appear in some other set in $\mathcal {C}$ that precedes $C_i$ in the labelling order. This forces $C_i$ to contain $n+i-1$ *uniquely*, because *N* is tree-child (and so each set in the cover must contain a uniquely appearing element). As an additional consequence of $n+j$ appearing twice in $\mathcal {C}$, we know that $|C_j|=1$, by Theorem 4.6. Then, by the first part of this Theorem statement, as $C_j$ is a singleton, it must be the first element of a pair in Category 1.

Finally, observe that if we have some pair in Category 1, say $C_j,C_{j+1}$, then $n+j$ is contained in $C_{j+1}$ due the spinal condition, and due to Theorem 4.6 is repeated in some subsequent set $C_i$. In $C_i$ there must be a unique element, implying that the remaining element, necessarily $n+i-1$, is unique. This is exactly the conditions required for a set in Category 3. The theorem follows. $\square$

Recall the following classes of interior vertices on a spine (noting that it runs from a leaf to the root), which were informally defined while outlining the proof strategy in Sect. 4.2.

#### Definition 4.8

Let *v* be an interior vertex or root vertex of a binary spinal network. Each such vertex will be an endpoint of one arc that is not part of the spinal path, leading to some vertex *w*, which we term the *non-spinal adjacent vertex* of *v*.

- If *w* is a leaf, we call *v* an *L*-vertex;
- if *w* is a vertex of the spine prior to *v* we call *v* a *P*-vertex; and
- if *w* is a vertex of the spine subsequent to *v* we call *v* an *R*-vertex.

We can therefore map each binary spinal network to a finite sequence of the letters $\{L,R_1,\dots ,R_k,P_1,\dots ,P_k\}$ containing $n-1$ *L*’s and 1 each of the remaining letters, which we term an *LRP-sequence*.

Recall that by Lemma 3.1 both the spine and spinal path are either unique or, if the internal vertex *v* farthest from the root has two leaf children, unique up to choice of the first leaf. In the latter case, either leaf selected to be part of the spine will result in the non-spinal adjacent vertex being a leaf, and so *v* will be an *L*-vertex, leaving no ambiguity. Additionally, note that the non-spinal adjacent vertex cannot be the next or previous vertex of *v* along the spinal path, as we do not permit parallel arcs. With these definitions, we can equivalently re-state Theorem 4.7 as follows:

#### Theorem 4.9

Let *N* be a binary spinal TCN with expanding cover $\mathcal {C}$. Then a set $C_i\in \mathcal {C}$ falls into one of the following three mutually exclusive categories:

1. It is part of a consecutive pair of sets $C_i,C_{i+1}$ such that $C_i$ corresponds to an *R*-vertex and $C_{i+1}$ corresponds to an *L*-vertex; or
2. $C_i$ corresponds to an *L*-vertex and $C_{i-1}$ does not correspond to an *R*-vertex; or
3. $C_i$ corresponds to a *P*-vertex that has a non-spinal arc to the first element of a pair in Category 1.

Indeed, for each pair of sets in Category 1, there is a corresponding set in Category 3.

#### Observation 4.10

Considering the rules of the LRP-sequences in the proof sketch in Sect. 4.2, the characterisation presented in Theorem 4.9 is equivalent to adding a new rule (the first of three we will introduce): **(R4a)** $R_i$ must always be followed immediately by *L*. This rule is a direct consequence of the first category in Theorem 4.9, as this is the only category in which *R*-vertices appear.

Our enumeration proof will therefore count how many ways to arrange sub-subsequences of the forms $R_iL,L,P_i$ in which there are *k* of the form $R_iL$, $n-1-k$ of the form *L*, and *k* of the form $P_i$, to make our subsequence, while still enforcing the rules mentioned in Sect. 4.2. This will be achieved by mapping to an appropriate set.

Denote the set of binary spinal TCNs on *n* leaves with *k* reticulations by $\mathcal {STC}_{n,k}$, and the set of expanding covers of such networks by $C(\mathcal {STC}_{n,k})$. Denote the set of partitions of $n+k$ into $n-k$ sets of size 1 and *k* sets of size 2 by $\mathcal {B}_{n,k}$. The sequence $|\mathcal {B}_{n-1,k}|$ is well-known, appearing off-by-one (i.e. as $|\mathcal {B}_{n,k}|$) as sequence A001498 (OEIS Foundation Inc 2024), the coefficients of the Bessel polynomial, and having exact formula

5 $$\begin{aligned} |\mathcal {B}_{n-1,k}| = \frac{(n-1+k)!}{2^k(n-1-k)!k!}. \end{aligned}$$

Finally, recall the well-known fact that for a TCN on *n* leaves, we must have $k \le n-1$ reticulations (see, for example, Cardona et al. (2008)).

#### Theorem 4.11

Let $\mathcal {STC}_{n,k}$ be the set of binary spinal TCNs on *n* leaves with $k \le n-1$ reticulations. Then, if $n>1$,

6 $$\begin{aligned} |\mathcal {STC}_{n,k}|=n!|\mathcal {B}_{n-1,k}|-\frac{n!}{2}|\mathcal {B}_{n-2,k}|, \end{aligned}$$

where $$|\mathcal {B}_{0,k}|=0$$. Equivalently,

7 $$\begin{aligned} |\mathcal {STC}_{n,k}|=\frac{n!(n-2+k)!(n-1+3k)}{2^{k+1}k!(n-1-k)!}. \end{aligned}$$

#### Proof

We proceed by finding a surjection $\varphi$ between $C(\mathcal {STC}_{n,k})$ and $\mathcal {B}_{n-1,k}$ that will allow us to enumerate $\mathcal {STC}_{n,k}$.

Suppose we have a binary spinal TCN *N*. By Theorem 4.9, together with R1–R3, *N* has an associated LRP-sequence, consisting of subsequences of the form $R_iL$, *L*, and $P_i$, that follow R1–R3 (noting that R3 is immediately satisfied by any such sequence since every $R_i$ is always followed by *L*). In particular, as noted in Observation 4.10, there are *k* subsequences of the form $R_iL$, $n-1-k$ subsequences of the form *L*, and *k* subsequences of the form $P_i$, for a total of $n-1+k$ subsequences. Each of these subsequences occupies some position $j \in [n-1+k]$, where positions are numbered from left to right.

We map valid LRP-sequences to elements of $\mathcal {B}_{n-1,k}$ via a mapping $\varphi$ defined as follows. For each *i*, let *p* be the position of the subsequence $R_iL$ and let *q* be the position of the corresponding $P_i$. By R2 we have $p<q$, and we associate to this pair the 2-element part $\{p,q\}$. Repeating this for all $i=1,\dots ,k$ yields *k* such parts. For every subsequence *L* occurring at position *r*, we associate the singleton part $\{r\}$. Hence $\varphi$ produces a partition of $[n-1+k]$ into *k* parts of size 2 and $n-1-k$ parts of size 1, i.e. an element of $\mathcal {B}_{n-1,k}$.

It remains to note that $\varphi$ is a bijection. Injectivity follows because the image uniquely determines which positions form $R_iL$–$P_i$ pairs and which correspond to singletons. Surjectivity follows since any partition in $\mathcal {B}_{n-1,k}$ may be realized by placing $R_iL$ at the smaller element of each 2-block, $P_i$ at the larger element, and singletons *L* at the positions of the 1-blocks, which satisfies R1–R3. Thus $\varphi$ is a bijection between the set of valid LRP-sequences for binary spinal TCNs and $\mathcal {B}_{n-1,k}$.

However, note that this bijection was determined entirely independently of leaf labels, and thus the image of an element under $\varphi$ is only unique up to tree shape. It follows that $\mathcal {B}_{n-1,k}$ counts exactly the network shapes of elements of $\mathcal {STC}_{n,k}$, and to find $|\mathcal {STC}_{n,k}|$ we may appeal to Lemma 4.1, resulting in the statement of the theorem. We can find the equivalent direct formulation by substituting in Equation (5) appropriately. $\square$

**Fig. 7:**
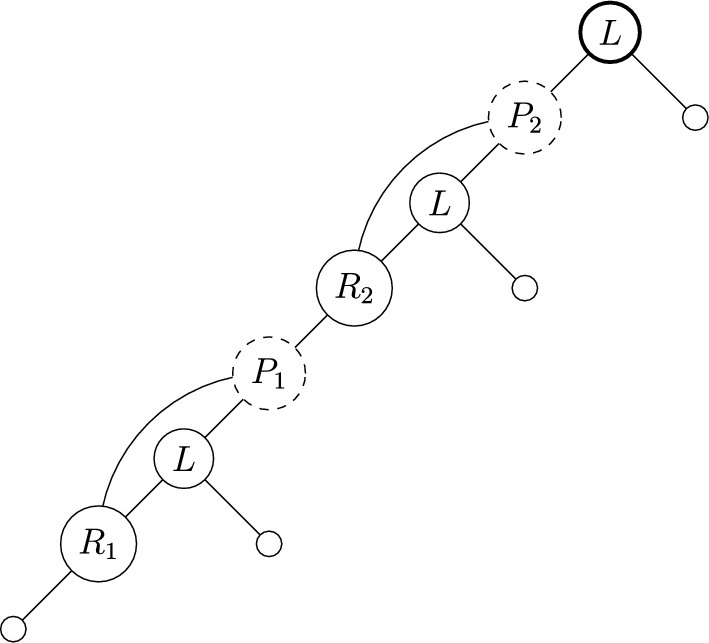
An example of a binary spinal tree-child network with interior vertices labelled according to the associated LRP-sequence. Each vertex is drawn according to which category they are in. Category 1 are plain circles, Category 2 are bold, and Category 3 are dashed. Refer to Example 4.12 for more detail

#### Example 4.12

Take the network shape depicted in Figure 7. This has the associated LRP-sequence $(R_1,L,P_1,R_2,L,P_2,L)$. Underlining to group together elements in the subsequences provided in the above proof, together with a subscript to indicate position, this is $(\underline{R_1,L}_1,\underline{P_1}_2,\underline{R_2,L}_3,\underline{P_2}_4,\underline{L}_5)$. Here we can see that $R_1L$ and $P_1$ are in position 1 and 2 respectively, giving the part $\{1,2\}$. Similarly, $R_2L$ and $P_2$ are in positions 3 and 4, giving the part $\{3,4\}$. Finally, the remaining *L* is in position 5, giving the singleton $\{5\}$. Therefore, the partition this network shape is mapped to is 12|34|5.

The sequence $|\mathcal {STC}_{n,k}|$ does not appear to be in the OEIS, so we have included some small values in Table 3. However, as $k \le n-1$ due to $\mathcal {STC}_{n,k}$ consisting of tree-child networks, we can find further associated sequences. In particular, we have the following results.

#### Theorem 4.13

Let $\mathcal {STC}_n$ be the set of binary spinal TCNs on *n* leaves, and let $\mathcal {STCS}_n$ be the set of shapes of binary spinal TCNs on *n* leaves. Then

8 $$\begin{aligned} |\mathcal {STC}_n| = \sum _{k=0}^{n-1} |\mathcal {STC}_{n,k}|, \end{aligned}$$

and

$$\begin{aligned} |\mathcal {STCS}_n| = \sum _{k=0}^{n-1} |\mathcal {B}_{n-1,k}|. \end{aligned}$$

The latter of these two sequences appears in the OEIS as A001515 (OEIS Foundation Inc 2024), which simply records the sums of Bessel numbers, while the former does not appear to have a sequence in the OEIS. The first elements of the sequence $|\mathcal {STC}_n|$ can be found in the Total row in Table 3.

**Table 3:** Values for $|\mathcal {STC}_{n,k}|$ where $1 \le n \le 8$ and $0 \le k < n$. Note, the row for $k=0$ counts caterpillar trees, and the row for $k=1$ counts all binary spinal networks with one reticulation, since any network with one reticulation is tree-child (given in the $k=1$ row of Table 9). Empty cells are zero counts

$k\backslash n$	**1**	**2**	**3**	**4**	**5**	**6**	**7**	**8**
**0**	1	1	3	12	60	360	2520	20160
**1**		2	15	108	840	7200	68040	705600
**2**			18	324	4500	59400	793800	11007360
**3**				360	11700	264600	5292000	101606400
**4**					12600	642600	21432600	603288000
**5**						680400	50009400	2305195200
**6**							52390800	5239080000
**7**								5448643200
**Total**	1	3	36	804	29700	1654560	129989160	13709545920

**Fig. 8:**
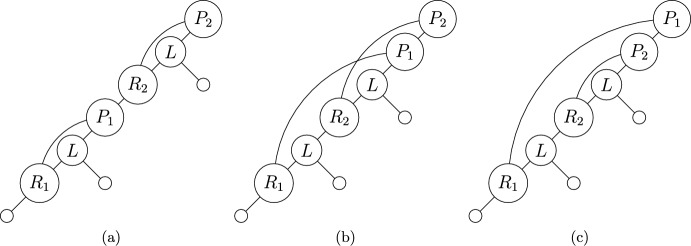
The three binary spinal TCNs with 3 leaves and 2 reticulations, up to labelling of the leaves

We depict the 3 binary spinal TCN shapes for $n=3,k=2$ in Figure 8. The $n=3,k=2$ entry of Table 3 can then be retrieved by making the 3! possible leaf labellings for each of them, for a total of $3 \times 3! = 18$ TCNs. We also make the following observation regarding caterpillar trees.

#### Observation 4.14

The set of binary spinal tree-child networks on *n* leaves with 0 reticulations is exactly the set of caterpillar trees on *n* leaves. By setting $k=0$ in Equation (5), we see that $B_{n,0} =1$ for all *n*, and so can, by Theorem 4.11 (or by directly substituting into Equation (7)), retrieve the well-known fact that the number of caterpillar trees on *n* leaves is exactly *n*!/2 (see, for example, (Steel 2016, p11)).

### The Class of Binary Spinal Stack-Free Networks

The enumeration of binary spinal stack-free networks proceeds in a similar manner to that for binary spinal tree-child networks. Recall the following definition.

#### Definition 4.15

A network is *stack-free* if every child of a reticulation vertex is a tree vertex.

Examples for $n=2$ leaves and $k=2$ reticulations are shown in Figure 10. We begin with an analogous result to Theorem 4.9, which is proved in a very similar way and so the proof is omitted.

#### Theorem 4.16

Let *N* be a binary spinal stack-free network with expanding cover $\mathcal {C}$. Then a set $C_i\in \mathcal {C}$ falls into one of the following four mutually exclusive categories:

1. It is part of a consecutive pair of sets $C_i,C_{i+1}$ such that $C_i$ corresponds to an *R*-vertex and $C_{i+1}$ corresponds to an *L*-vertex; or
2. It is part of a consecutive pair of sets $C_i,C_{i+1}$ such that $C_i$ corresponds to an *R*-vertex and $C_{i+1}$ corresponds to a *P*-vertex that has an arc to the first element of a pair in Categories 1 or 2 that is not $C_i$; or
3. $C_i$ corresponds to an *L*-vertex and $C_{i-1}$ is not an *R*-vertex; or
4. $C_i$ corresponds to a *P*-vertex that has an arc to the first element of a pair in Categories 1 or 2 and $C_{i-1}$ is not an *R*-vertex.

Indeed, for each pair of sets in Category 1, there is a corresponding *P*-vertex in Category 2 or 4.

#### Observation 4.17

Considering the rules of the finite sequences in the proof sketch in Sect. 4.2, this is equivalent to adding a new rule that is a relaxed form of the rule for tree-child networks: **(R4b)** $R_i$ may be followed immediately by *L* or $P_j$ for $i \ne j$, but cannot be followed immediately by any $R_j$. Our enumeration proof will therefore count how many ways to arrange subsequences of the forms $R_iL,R_iP_j,L,P_i$ in which, for some $0<m<k$ there are *m* of the form $R_iL$, $k-m$ of the form $R_iP_j$, $n-1-m$ of the form *L* and *m* of the form $P_i$, to make our sequence, while still enforcing the rules mentioned in Sect. 4.2. This will be achieved by mapping to an appropriate set.

Denote the set of partitions of a set of *n* distinct elements into *k* parts by $S_2(n,k)$, noting that these are counted by the Stirling numbers of the second kind. A closed formula for these exists, see entry A008277 on the OEIS (OEIS Foundation Inc 2024), namely

9 $$\begin{aligned} |S_2(n,k)| = \sum _{j=0}^k \frac{(-1)^{k-j}j^n}{(k-j)!j!}. \end{aligned}$$

We can now show the main result of this section. Let $\mathcal {SSF}_{n,k}$ be the class of binary spinal stack-free networks with *n* leaves and *k* reticulations, and the set of expanding covers of such networks be $C(\mathcal {SSF}_{n,k})$. Then we have the following.

#### Theorem 4.18

The cardinality of the class of binary spinal stack-free networks with *n* leaves and *k* reticulations, $\mathcal {SSF}_{n,k}$, is

$$\begin{aligned} |\mathcal {SSF}_{n,k}|=n!\left| S_2(n-1+k,n-1)\right| -\frac{n!}{2}|S_2(n-2+k,n-2)|. \end{aligned}$$

Equivalently,

10 $$\begin{aligned} |\mathcal {SSF}_{n,k}|=n(n-1)^{n-1+k} - \frac{n!}{2} \sum _{j=0}^{n-2} \frac{(-1)^{n-j}(n-1+j)j^{n-2+k}}{(n-1-j)!j!}. \end{aligned}$$

#### Observation 4.19

As with the case of tree-child networks, note that when $k=0$, these expressions reduce to $\frac{n!}{2}$, which counts the number of caterpillar trees on *n* leaves (this is easiest to see in the first expression, because $S_2(n-1,n-1)=1$).

#### Proof

We intend to construct a surjection from $C(\mathcal {SSF}_{n,k})$ to $S_2(n-1+k,n-1)$ that will allow us to enumerate $\mathcal {SSF}_{n,k}$.

Suppose we have a binary spinal TCN *N*. By Theorem 4.16, together with R1–R3, *N* has an associated LRP-sequence consisting of subsequences of the form $R_iL$, $R_iP_j$, *L*, and $P_i$, which satisfy R1–R3. In particular, as noted in Observation 4.17, for some $0<m<k$ there are *m* subsequences of the form $R_iL$, $k-m$ of the form $R_iP_j$, $n-1-m$ of the form *L*, and *m* of the form $P_i$, giving a total of $n-1+k$ subsequences. Each subsequence occupies a position $j \in [n-1+k]$, where positions are numbered from left to right.

Define an equivalence relation $\sim$ on $[n-1+k]$ in the following way:

1. $a \sim a$ for all $a \in [n-1+k]$; and
2. $a \sim b$ if *a* is the position of an $R_iL$-subsequence with a corresponding $P_i$- or $R_jP_i$-subsequence at position *b*, or vice versa.
3. $a_i \sim a_j$ if there exists a chain of relations in 2) of the form $a_i \sim a_{i+1},\dots ,a_{j-1} \sim a_j$.

This equivalence relation partitions $[n-1+k]$, and we claim that the induced partition determines a bijection from LRP-sequences of networks in $\mathcal {SSF}_{n,k}$ to $S_2(n-1+k,n-1)$.

Let *a* denote the number of subsequences of the form $R_iL$ and *b* the number of subsequences of the form *L*. Since each leaf not on the spine is represented exactly once, there is a one-to-one correspondence between these subsequences and the $n-1$ non-spinal leaves, and hence $a+b = n-1$. Moreover, the subsequences that occupy the least position in each $\sim$-part must be of the form $R_iL$ or *L*, so the number of $\sim$-parts is also $a+b = n-1$. Thus the equivalence relation induces a partition of $[n-1+k]$ into $n-1$ parts.

We note that the original LRP-sequence can be uniquely retrieved, as the position of *L* subsequences can be immediately found from the singletons in a given partition, and the positions of each of the remaining types of subsequences can be retrieved by ordering the elements within a part. For a given part, the lowest element corresponds to the position of a subsequence of the form $R_iL$, the intermediate elements correspond to the position of a subsequence of the form $R_iP_j$, and the largest element corresponds to the position of a subsequence of the form $P_i$.

The partition induced by $\sim$ is uniquely determined by the network up to permutation of the leaves. Consequently, the number of network shapes in $\mathcal {SSF}_{n,k}$ is given by $|S_2(n-1+k,n-1)|$, which is sequence A354977 (offset by 1 in both parameters of $S_2$, i.e. $|S_2(n+k,n)|$) in the OEIS (OEIS Foundation Inc 2024). By Lemma 4.1, this establishes the first statement of the theorem. The closed form in Equation (10) then follows from Equation (9) after a straightforward algebraic manipulation. $\square$

#### Example 4.20

**Fig. 9:**
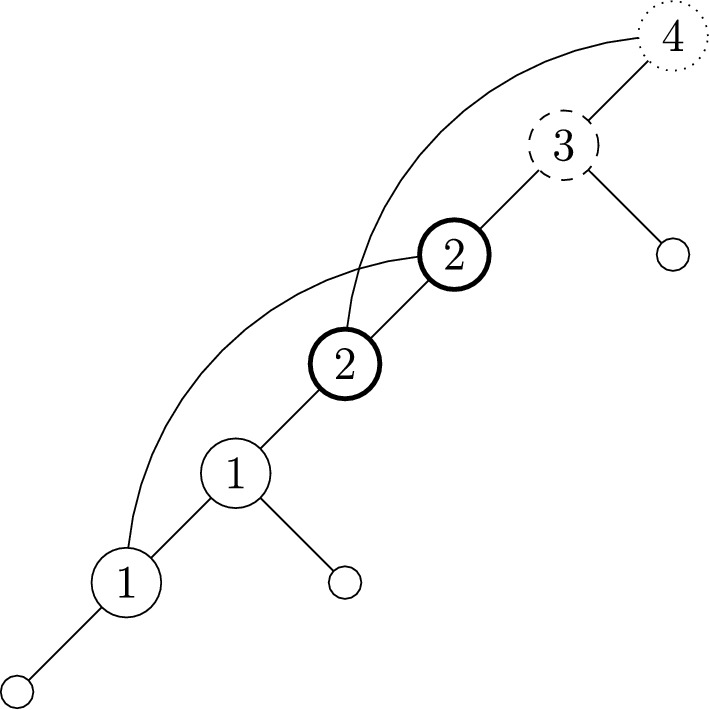
An example of a binary spinal stack-free network with interior vertices labelled according to the position of their associated subsequence in the sequence described in Theorem 4.18. Each vertex is drawn according to which category they are in. Category 1 are plain circles, Category 2 are dashed, Category 3 are bold, and Category 4 are dotted. This example is mapped to the partition 124|3

Take the network shape depicted in Figure 9. This has the associated LRP-sequence $(R_1,L,R_2,P_1,L,P_2)$. Underlining to group together elements in the subsequences provided in the above proof, together with a subscript to indicate position, this is $(\underline{R_1,L}_1,\underline{R_2,P_1}_2,\underline{L}_3,\underline{P_2}_4)$. The equivalence relation gives $1 \sim 2$ and $2 \sim 1$ (as $R_1$ is in position 1 and its corresponding parent $P_1$ is in position 2), and similarly gives $2 \sim 4$ and $4 \sim 2$. Rule 3) of the equivalence relation then gives $1 \sim 4$ and $4 \sim 1$. Finally, 3 is only related to itself. Thus the equivalence relation precisely gives the partition 124|3.

**Fig. 10:**
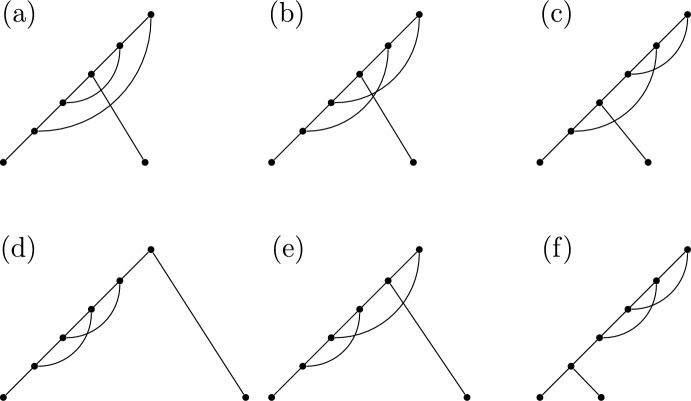
All binary spinal network shapes with $n=2$ leaves and $k=2$ reticulations. Networks (a) and (b) are fully tree-sibling but not stack-free, while (c) is stack-free but not fully tree-sibling. Networks (d), (e), and (f) are neither fully tree-sibling or stack-free. This can be seen by observing that (a), (b) and (d-f) each contain a reticulation vertex with a reticulation vertex child, and so are not stack-free. Similarly, in (c-f), the two reticulation vertices are siblings, and hence these networks are not fully tree-sibling. Note, there are no tree-child networks with $n=k=2$ (see Table 3). There are two leaf-labelled copies of each of shapes (a) to (e), while (f) has only one

The sequence corresponding to Equation (10) does not appear to be in the OEIS, so we have included some small values in Table 4. One can see the unique network shape for $n=2,k=2$ in Figure 10(c), and retrieve the corresponding entry of 2 in Table 4 by assigning leaf labels in the two possible ways.

Binary spinal stack-free networks do not have a limit on the number of reticulations that may appear for fixed $n>1$. To see this, we note that we can construct a valid LRP-sequence corresponding to a binary spinal stack-free network on two leaves with *k* reticulations for any $k \in \mathbb {N}$. This is done by starting with $R_1L$, followed by $R_2P_1R_3P_2 \dots R_kP_{k-1}$, followed by a final $P_k$. This construction may be extended to any number of leaves *n* by simply appending $n-2$ *L*s to the end of the sequence. Hence, there exists a binary spinal stack-free network on *n* leaves with *k* reticulations for any $k \in \mathbb {N}$, and so we cannot achieve an analogous result to the previous section where we found an expression for $|\mathcal {STC}_n|$ by summing over all *k*.

**Table 4:** The number of binary spinal stack-free networks $|\mathcal {SSF}_{n,k}|$, for $2 \le n \le 8$ and $0 \le k \le 8$, obtained using Theorem 4.18

$k\backslash n$	**2**	**3**	**4**	**5**	**6**	**7**	**8**
**0**	1	3	12	60	360	2520	20160
**1**	2	15	108	840	7200	68040	705600
**2**	2	39	516	6300	77400	987840	13265280
**3**	2	87	1980	36600	630000	10689840	183738240
**4**	2	183	6852	186060	4392360	97531560	2119763520
**5**	2	375	22428	874440	27820800	797451480	21678148800
**6**	2	759	71076	3911100	165367800	6049446480	203761071360
**7**	2	1527	220860	16930200	940698000	43503324480	1801038919680
**8**	2	3063	677892	71670060	5185980360	300797897400	15201573266880

### The Class of Binary Spinal Fully Tree-Sibling Networks

The tree-child result in Equation (6) can be extended to “fully tree-sibling” networks, which we introduce here.

#### Definition 4.21

A network *N* is *fully tree-sibling* if and only if every sibling of a reticulation vertex is a tree vertex.

The class of fully tree-sibling networks, denoted $\mathcal {FTS}_{n,k}$, generalises the class of tree-child networks, as stacks are permitted, but are a subclass of tree-sibling networks, as tree-sibling networks only require each reticulation vertex to have at least one sibling that is a tree vertex. Some examples are shown in Figure 10.

Our intent is now to enumerate binary spinal fully tree-sibling networks. As the proof for this theorem is very similar to the previous proofs for the corresponding theorems in the previous sections, the proof is omitted.

#### Theorem 4.22

Let *N* be a binary spinal fully tree-sibling network with expanding cover $\mathcal {C}$. Then a set $C_i\in \mathcal {C}$ falls into one of the following three mutually exclusive categories:

1. It is part of a consecutive sequence of sets $C_i,\dots ,C_{h}$ such that $C_i,\dots ,C_{h-1}$ correspond to *R*-vertices and $C_{h}$ corresponds to an *L*-vertex; or
2. $C_i$ corresponds to an *L*-vertex and $C_{i-1}$ is not an *R*-vertex; or
3. $C_i$ corresponds to a *P*-vertex that has an arc to some *R*-vertex of a sequence in Category 1.

Indeed, for each sequence of sets of length $h-1$ in Category 1, there are $h-1$ corresponding *P*-vertices in Category 3.

#### Observation 4.23

Considering the rules of the finite sequences in the proof sketch in Sect. 4.2, this is equivalent to adding a new rule that is a relaxed form of the rule for tree-child networks: **(R4c)** $R_i$ may be followed immediately by *L* or $R_j$ for $i < j$, but cannot be immediately followed by any $P_j$. Our enumeration proof will therefore count how many ways to arrange subsequences of the forms $R_i\dots R_hL,L,P_i$ in which, for some $0< m \le k$ there are *m* of the form $R_i\dots R_hL$, $n-1-m$ of the form *L* and *k* of the form $P_i$, to make our sequence, while still enforcing the rules mentioned in Sect. 4.2. This will be achieved by mapping to an appropriate set.

Denote the set of permutations of a set of *n* distinct elements that can be written in reduced form using *c* disjoint cycles, by $S_1(n,c)$ — the Stirling numbers of the first kind. We can now show the main result of this section. Let $\mathcal {FTS}_{n,k}$ be the class of fully tree-sibling binary spinal networks on *n* leaves with *k* reticulations, and $C(\mathcal {FTS}_{n,k})$ be the set of expanding covers corresponding to these networks. Then we have the following.

#### Theorem 4.24

The cardinality of the class of fully tree-sibling binary spinal networks on *n* leaves with *k* reticulations, $\mathcal {FTS}_{n,k}$, is

11 $$\begin{aligned} \left| \mathcal {FTS}_{n,k}\right| =n!\left| S_1(n-1+k,n-1)\right| -\frac{n!}{2}|S_1(n-2+k,n-2)|. \end{aligned}$$

#### Proof

We intend to construct a surjection from $C(\mathcal {FTS}_{n,k})$ to $S_1(n-1+k,n-1)$ that will allow us to enumerate $\mathcal {FTS}_{n,k}$.

Suppose we have a binary spinal TCN *N*. By Theorem 4.22, together with R1–R3, *N* has an associated LRP-sequence consisting of subsequences of the form $R_i\dots R_hL,L,P_i$, which satisfy R1–R3. In particular, as noted in Observation 4.23, for some $0< m \le k$ there are *m* of the form $R_i\dots R_hL$, $n-1-m$ of the form *L* and *k* of the form $P_i$, giving a total of $n-1+k$ subsequences. Each subsequence occupies a position $j \in [n-1+k]$, where positions are numbered from left to right.

We construct a surjection from the set of LRP-sequences of networks in $\mathcal {FTS}_{n,k}$ to $S_1(n-1+k,n-1)$ in the following way. Subsequences of the form *L* are mapped to singleton cycles of their corresponding position label. Subsequences of the form $R_i\dots R_hL$ together with the corresponding *h* subsequences of the form $P_i$ are mapped to $h+1$-cycles in which the first element is the position label of $R_i\dots R_hL$, and the remaining elements are the labels of the corresponding $P_i$-subsequences, ordered according to the index *i* (that is, the label of $P_i$ is first, then the label of $P_{i+1}$, etc.).

We now claim there will be $n-1$ such cycles. This follows directly from the fact that we produce a cycle exactly once for each subsequence of the form $R_i\dots R_hL$ or *L*, of which there are precisely $n-1$ total.

One can retrieve the initial LRP-sequence directly. Singleton cycles correspond to the positions of *L* subsequences. For the remaining types the lowest element in each cycle corresponds to the position of a $R_i\dots R_hL$ subsequence, and the remaining elements in the cycle correspond to the position of $P_i$ subsequences, with indices retrieved according the ordering in the cycle.

It is again clear that the mapped permutation is unique for each network up to permutation of the leaves, and hence the number of network shapes in $\mathcal {FTS}_{n,k}$ is $|S_1(n-1+k,n-1)|$, which appears in the OEIS (off by 1 in both parameters of $S_1$, i.e. $|S_1(n+k,n)|$) as A354979 (OEIS Foundation Inc 2024). By Lemma 4.1, we obtain the statement of the theorem. $\square$

The sequence corresponding to Equation (11) does not appear to be in the OEIS, so we have included some small values in Table 5. One can see the two possible tree shapes for $n=2,k=2$ in Figure 10 (a) and (b), and retrieve the corresponding entry of 4 in Table 4 by assigning leaf labels in the two possible ways for each.

**Table 5:** The number of binary spinal fully tree-sibling networks $|\mathcal {FTS}_{n,k}|$, for $2 \le n \le 8$ and $0 \le k \le 8$, using Theorem 4.24

$k\backslash n$	**2**	**3**	**4**	**5**	**6**	**7**	**8**
**0**	1	3	12	60	360	2520	20160
**1**	2	15	108	840	7200	68040	705600
**2**	4	60	708	8100	95400	1181880	15523200
**3**	12	282	4800	74700	1146600	17922240	289578240
**4**	48	1572	35688	714840	13726440	262324440	5085823680
**5**	240	10224	294000	7286160	169691760	3867658200	88160909760
**6**	1440	76248	2678160	79754160	2199664800	58620592800	1545081088320
**7**	10080	642384	26829792	938778000	30089505600	922684316400	27736453704960
**8**	80640	6038496	293766912	11865754560	435296014560	15157877454720	513727745731200

#### Example 4.25

**Fig. 11:**
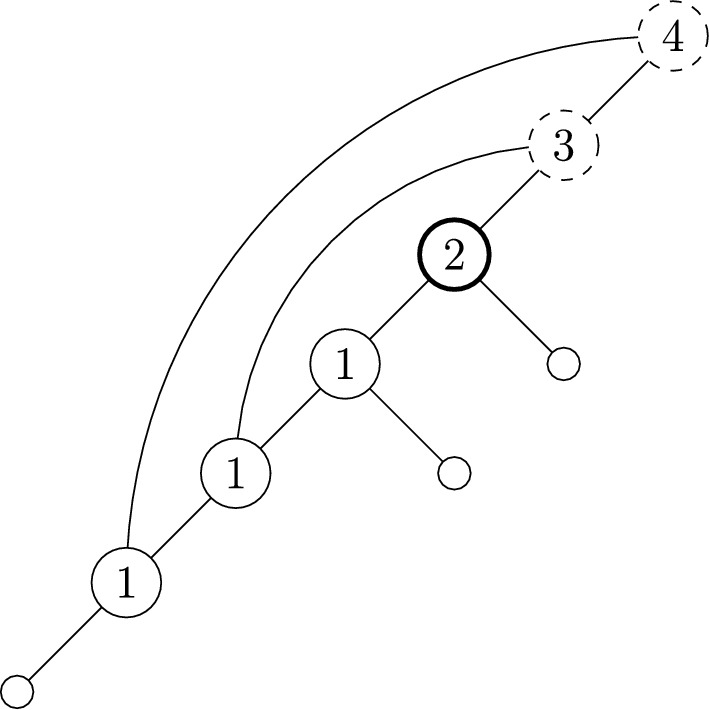
An example of a binary spinal fully tree-sibling network with interior vertices labelled according to the position of their associated subsequence in the sequence described in Theorem 4.24. Each vertex is drawn according to which category they are in. Category 1 are plain circles, Category 2 are dashed, and Category 3 are bold. This example is mapped to, in cycle notation, (143)(2)

Take the network shape depicted in Figure 11. This has the associated LRP-sequence $(R_1,R_2,L,L,P_2,P_1)$. Underlining to group together elements in the subsequences provided in the above proof, together with a subscript to indicate position, this is $(\underline{R_1,R_2,L}_1,\underline{L}_2,\underline{P_2}_3,\underline{P_1}_4)$. Under the mapping, the *L* in position 2 maps to the singleton (2). The $R_1R_2L$-subsequence is in position 1, the parent of $R_1$ is in position 4 and the parent of $R_2$ is in position 3. This gives the cycle (143). Putting this together, this network shape corresponds to the permutation (143)(2).

Binary spinal fully tree-sibling networks do not have a limit on the number of reticulations that may appear. for fixed $n>1$. To see this, we note that we can construct a valid LRP-sequence corresponding to a binary spinal fully tree-sibling network on *n* leaves with *k* reticulations for any $k \in \mathbb {N}$, for instance the sequence $R_1\dots R_k L P_k \dots P_1 L \dots L$, where the *L*’s on the suffix are repeated $n-2$ times. Hence, there exists a binary spinal stack-free network on *n* leaves with *k* reticulations for any $k \in \mathbb {N}$, and so we cannot achieve an analogous result to Sect. 4.3 where we found an expression for $|\mathcal {STC}_n|$ summing over all *k*.

#### Summary of LRP Rules

We have used sets of LRP rules to define different subclasses of spinal networks throughout Sections 4.3, 4.4, and 4.5, and summarise them in Table 6. Note that the class $\mathcal {STC}_{n,k}$ follows all six given rules, as Rule 4a implies Rules 4b and 4c.

**Table 6:** A summary of the LRP rules for the network classes described in Sections 4.3, 4.4, and 4.5

		**Network Class**		
**Rule**	**Description**	$\mathcal {STC}$	$\mathcal {SSF}$	$\mathcal {FTS}$
**1**	If $j>i$, then $R_j$ must appear subsequently to $R_i$	$\checkmark$	$\checkmark$	$\checkmark$
**2**	$P_i$ must appear subsequently to $R_i$	$\checkmark$	$\checkmark$	$\checkmark$
**3**	$P_i$ must not immediately follow $R_i$	$\checkmark$	$\checkmark$	$\checkmark$
**4a**	$R_i$ must always be followed immediately by *L*	$\checkmark$	$\times$	$\times$
**4b**	$R_i$ cannot be followed immediately by any $R_j$	$\checkmark$	$\checkmark$	$\times$
**4c**	$R_i$ cannot be immediately followed by any $P_j$	$\checkmark$	$\times$	$\checkmark$

### Other Classes

#### Parallel Arcs Allowed

Consider the class of binary spinal networks on *n* leaves with *k* reticulations with no further restrictions, and allow parallel arcs. Then, up to rearrangement of the leaves, this will be equivalent to labelling the internal vertices from 1 to $n-1+2k$, and then selecting *k* pairs of internal vertices and for each pair, say *i* and *j* with $i<j$, set the vertex labelled *i* to be the non-spinal child of the vertex labelled *j*. Enumerating the class is therefore simply the number of ways of selecting *k* pairs of numbers from 1 to $n-1+2k$, which is

$$\begin{aligned} \frac{\left( {\begin{array}{c}n-1+2k\\ 2\end{array}}\right) \left( {\begin{array}{c}n-3+2k\\ 2\end{array}}\right) \dots \left( {\begin{array}{c}n+1\\ 2\end{array}}\right) }{k!} = \frac{(n-1+2k)!}{2^k(n-1)!k!}. \end{aligned}$$

This is a standard combinatorial identity, and the values can be found amongst the sequence A100861 on the OEIS (OEIS Foundation Inc 2024), after a suitable variable shift. In particular, as the OEIS sequence counts, among other things, the number of ways of selecting *k* pairs from 1 to *n*, these values can be found as sequence element $(n',k)$, where $n'=n-1+k$. Then, by Lemma 4.1, we have the following result.

##### Theorem 4.26

Let $\mathcal {BSP}_{n,k}$ be the class of binary spinal networks, in which parallel arcs are permitted. Then

12 $$\begin{aligned} {\begin{matrix} |\mathcal {BSP}_{n,k}| & =n!\left( \frac{(n-1+2k)!}{2^k(n-1)!k!}-\frac{(n-2+2k)!}{2^{k+1}(n-2)!k!}\right) \\ & = \frac{n(n-1+4k)(n-2+2k)!}{2^{k+1}k!}. \end{matrix}} \end{aligned}$$

**Fig. 12:**
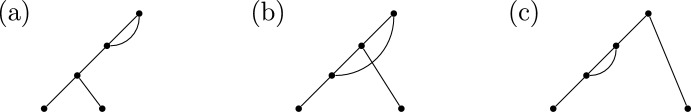
All binary spinal network shapes with parallel edges allowed with $n=2$ leaves and $k=1$ reticulations. Note that (b) is the only one of these three that does not contain a parallel edge, and so with assignment of leaf labels corresponds to the $n=2,k=1$ entry of 2 in Tables 3, 4, 5 and 9

We provide some small values in Table 7. We present the three binary spinal network shapes with parallel arcs permitted with 2 leaves and 1 reticulation in Figure 12, which corresponds to the entry 5 for $n=2,k=2$ in Table 7, which can be retrieved by assigning leaf labels in each of the two possible ways for Figure 10 (b) and (c), and in the one possible way for Figure 10 (a).

We note, again, that the class of binary spinal networks with parallel edges allowed does not have a limit on the number of reticulations that may appear, so there are infinitely many for each *n*, and so we cannot achieve an analogous result to Sect. 4.3 where we found an expression for $|\mathcal {STC}_n|$ summing over all *k*.

**Table 7:** Values for $\mathcal {BSP}_{n,k}$ where $1 \le n,k \le 8$

$k \backslash n$	**1**	**2**	**3**	**4**	**5**	**6**	**7**	**8**
**1**	1	5	27	168	1200	9720	88200	887040
**2**	3	27	225	1980	18900	196560	2222640	27216000
**3**	15	195	2205	25200	302400	3855600	52390800	758419200
**4**	105	1785	25515	359100	5197500	78586200	1248647400	20886465600
**5**	945	19845	343035	5738040	97297200	1702701000	30989158200	588453465600
**6**	10395	259875	5270265	102162060	1986484500	39502663200	810485676000	17228609798400
**7**	135135	3918915	91216125	2010808800	44108064000	982507125600	22438874858400	527973526080000
**8**	2027025	66891825	1757430675	43418875500	1060661101500	26162973837000	658316990331000	16987548201624000

#### Parallel Arcs Not Allowed

Enumeration of binary spinal networks on *n* leaves with *k* reticulations and no parallel arcs, denoted $\mathcal{B}\mathcal{S}_{n,k}$, is similar to the previous case, except that we may not select a consecutive pair of vertices. We constrast this to the result obtained in Theorem 4.2 for spinal networks with *n* leaves and $|\mathcal {C}|$ sets in the corresponding cover, by noting that in the present section we consider only binary networks, and we are restricting the number of reticulations rather than the number of sets in the corresponding cover.

Krasko and Omelchenko provide a recurrence relation in (Krasko and Omelchenko 2017, Lemma 2.2) for a related problem to that of enumeration of binary spinal networks with no parallel arcs, that obtains the same sequence. Consider $2n+2$ vertices arranged in a line, labelled $\{1, \dots , 2n+2\}$. An edge is then placed between pairs of vertices, referred to as a chord. If a chord connects two adjacent vertices with labels that differ by 1, the chord is referred to as a loop. Their result involves counting linear diagrams in which the number of loops is equal to a given value. Rather than construct a bijection between their construction and ours, we provide a direct proof that is adapted from theirs. Informally, we will follow a similar construction, considering the spine of a binary spinal network to be a linear diagram, and changing each loop into a single vertex with a leaf attached, in order to prevent parallel edges. This will count the number of network shapes, and a full count of networks may then be obtained using Lemma 4.1. Let $\mathcal {BSS}_{n,k}$ denote the class of shapes of binary spinal networks on *n* leaves with *k* reticulations and no parallel arcs. Additionally, let $\mathcal{B}\mathcal{S}_{n,k}$ denote the class of binary spinal networks on *n* leaves with *k* reticulations and no parallel arcs.

##### Theorem 4.27

The cardinality of the class of shapes of binary spinal networks on *n* leaves with *k* reticulations and no parallel arcs, $\mathcal {BSS}_{n,k}$, is

$$\begin{aligned} |\mathcal {BSS}_{n,k}| = |\mathcal {BSS}_{n-1,k}| + (n+2k-3)|\mathcal {BSS}_{n,k-1}| + n|\mathcal {BSS}_{n+1,k-2}|, \end{aligned}$$

where $|\mathcal {BSS}_{n,k}|=0$ for $k<0$, $|\mathcal {BSS}_{n,0}|=1$ for $n \ge 1$, and $|\mathcal {BSS}_{0,k}|=0$.

Additionally, the cardinality of the class of binary spinal networks on *n* leaves with *k* reticulations and no parallel arcs, $\mathcal{B}\mathcal{S}_{n,k}$, is

$$\begin{aligned} |\mathcal{B}\mathcal{S}_{n,k}|=n!|\mathcal {BSS}_{n,k}|-\frac{n!}{2}|\mathcal {BSS}_{n-1,k}|. \end{aligned}$$

##### Proof

We consider base cases first. There are no networks with zero leaves and no networks with a negative number of reticulations, so $|\mathcal {BSS}_{0,k}|=0$ and $|\mathcal {BSS}_{n,k}|=0$ for $k<0$. Clearly the only network shape in $\mathcal {BSS}_{n,0}$ is the caterpillar tree, so $|\mathcal {BSS}_{n,0}|=1$.

Consider a tree shape in $\mathcal {BSS}_{n,k}$. There are three possibilities for the final vertex *v* (the $n+2k$-th):

1. The non-spinal adjacent vertex of *v* is a leaf; or
2. The non-spinal adjacent vertex of *v* is a reticulation vertex *w*, and deletion of *v* and suppression of *w* would not result in a parallel arc; or
3. The non-spinal adjacent vertex of *v* is a reticulation vertex *w*, and deletion of *v* and suppression of *w* would result in a parallel arc.

We can thus construct the network shapes in $\mathcal {BSS}_{n,k}$ in the following three mutually exclusive ways:

1. Take a network shape in $\mathcal {BSS}_{n-1,k}$, add a new (root) vertex at the end of the spine, with a new leaf for its non-spinal adjacent vertex. For each given network shape, there is only one way to do this.
2. Take a network shape in $\mathcal {BSS}_{n,k-1}$, pick an arc of the path and subtend it to form a new vertex *w*. Then add a new (root) vertex *v* at the end of the path, and an arc from *v* to *w*. There are $n+2k-3$ arcs on the path, so $n+2k-3$ options for this.
3. Take a network shape in $\mathcal {BSS}_{n+1,k-2}$, and choose a vertex *w* for which the non-spinal adjacent vertex is one of the *n* leaves that are not on the spine. Delete this leaf, add a new (root) vertex *v* at the end of the path, and add an arc from *v* to *w*. Finally, subtend the two spinal arcs adjacent to *w*, and add an arc between the two new vertices created. There are *n* options for this.

From these observations, the first statement of the theorem follows. We obtain the final statement by application of Lemma 4.1. $\square$

Neither of the corresponding sequences from Theorem 4.27 appear to be in the OEIS, so we have included some small values in Tables 8 and 9. Note in particular that the only zero value in either table is the case $n=1,k=1$. In this case, one can see that no such network can exist, as a binary spinal network with one leaf and one reticulation has precisely 3 vertices, one of which is a leaf, so the reticulation must be the parent of the leaf and the parent of the reticulation along the spine must also be the parent of the reticulation along the non-spinal arc (and is the root), contradicting the fact that we are not allowing parallel arcs. Additionally, we present all six binary spinal network shapes with 2 leaves and 2 reticulations in Figure 10, which corresponds to the entry 6 for $n=2,k=2$ in Table 8. The corresponding entry in Table 9 of 11 can be retrieved by assigning leaf labels in each of the two possible ways for Figure 10 (a-e), and in the one possible way for Figure 10 (f).

**Table 8:** The number of binary spinal network shapes with no parallel arcs $|\mathcal {BSS}_{n,k}|$, for $1 \le n \le 7$, $0 \le k \le 8$, using Theorem 4.27

**k** $\backslash$ **n**	**1**	**2**	**3**	**4**	**5**	**6**	**7**
**0**	1	1	1	1	1	1	1
**1**	0	1	3	6	10	15	21
**2**	1	6	21	55	120	231	406
**3**	5	41	185	610	1645	3850	8106
**4**	36	365	2010	7980	25585	70371	172305
**5**	329	3984	25914	120274	446544	1410003	3932313
**6**	3655	51499	386407	2052309	8655780	30839655	96451278
**7**	47844	769159	6539679	39110490	184652985	732520998	2538187938
**8**	721315	13031514	123823305	823324755	4301276760	18797883390	71463760368

**Table 9:** The number of binary spinal networks with no parallel arcs $|\mathcal{B}\mathcal{S}_{n,k}|$, for $1 \le n \le 7$, $0 \le k \le 8$ using Theorem 4.27

**k** $\backslash$ **n**	**1**	**2**	**3**	**4**	**5**	**6**	**7**
**0**	1	1	3	12	60	360	2520
**1**	0	2	15	108	840	7200	68040
**2**	1	11	108	1068	11100	123120	1464120
**3**	5	77	987	12420	160800	2179800	31152240
**4**	36	694	10965	167400	2591400	41456520	691082280
**5**	329	7639	143532	2575608	46368840	854446320	16265649960
**6**	3655	99343	2163945	44618532	915555060	19088470800	408398510520
**7**	47844	1490474	36930597	860175612	19811728800	460940043960	10946514292560
**8**	721315	25341713	703845288	18273914460	466753725900	11986016407200	312806686111920

## Discussion

In this paper we have enumerated several classes of phylogenetic network that generalise the class of phylogenetic trees known as “caterpillar” trees. These trees are important mathematically, because they sit at one extreme of the space of rooted trees, as the least “balanced”, and their combinatorial properties are easier to handle than other trees. They are also of interest biologically, as in the presence of rapid evolution, such as with viral pathogens, the trees that arise are often close to caterpillar in shape (Fitch et al. 1991; Bush et al. 2000; Bryan et al. 2004). Analogously, we expect spinal networks to be of similar importance; a figure showing their place in the ecosystem of phylogenetic networks can be found in (Francis 2025, Fig. 4).

The subclasses of spinal networks that we have studied here have various containment relationships, as shown in Figure 3, and these inclusions can also be seen by tracking the numbers in various classes for fixed values of *n* and *k*. For instance, if we take $n=4$ and $k=2$ then we see

$$\begin{aligned} \text {Binary spinal tree-child networks } &  |\mathcal {STC}_{4,2}|&= 324\\ \text {Binary spinal stack-free networks } &  |\mathcal {SSF}_{4,2}|&= 516\\ \text {Binary spinal fully tree-sibling networks } &  |\mathcal {FTS}_{4,2}|&= 708\\ \text {Binary spinal networks } &  |\mathcal{B}\mathcal{S}_{4,2}|&= 1068\\ \text {Binary spinal networks (parallel allowed)} &  |\mathcal {BSP}_{4,2}|&= 1980. \end{aligned}$$

We can see that the ordering indicated by the Hasse diagram is respected:

$$|\mathcal {STC}_{n,k}|\le |\mathcal {FTS}_{n,k}| \le | \mathcal{B}\mathcal{S}_{n,k}|\le |\mathcal {BSP}_{n,k}|$$

and also

$$|\mathcal {STC}_{n,k}|\le |\mathcal {SSF}_{n,k}| \le | \mathcal{B}\mathcal{S}_{n,k}|.$$

Note that the class of all spinal networks $\mathcal {S}$ is indexed differently in this paper so the tables cannot be compared directly, while allowing parallel arcs in $\mathcal{S}\mathcal{P}$ results in an infinite class.

Phylogenetic networks pose many challenges with enumeration, because of their complexity, and so structural restrictions that provide the possibility of concrete results can be important. The present paper takes the approach of applying heavy restrictions on network structure, while taking advantage of a new framework for set-theoretic bijections with networks that have been recently developed (Francis et al. 2024; Francis and Steel 2023).

The use of covers for labellable networks may also help in finding recursive formulae for networks similar to the Pons-Batle conjecture in Eq. (1) (Pons and Batle 2021), and may provide new tools for further investigation of asymptotic properties of classes of network, along the lines of several recent developments (Fuchs et al. 2021, 2025).

Both these avenues seem interesting and important directions for future research.

## Data Availability

Not applicable.
